# Ovarian mucinous borderline tumor with anaplastic carcinomatous nodules in adolescents

**DOI:** 10.1186/s13048-022-01010-3

**Published:** 2022-07-14

**Authors:** Mengqi Huang, Qian Lv, Jingyan Xie

**Affiliations:** 1 Departments of Gynecology, Nanjing First Hospital, Nanjing Medical University, Nanjing, 210006 People’s Republic of China; 2 Departments of Pathology, Nanjing First Hospital, Nanjing Medical University, Nanjing, 210006 People’s Republic of China

**Keywords:** Adolescent, Ovarian tumor, Mural nodule, Pathological features, Treatment and prognosis

## Abstract

Bilateral ovarian epithelial neoplasms in adolescents are rare. Moreover, borderline mucinous neoplasms with local intraepithelial carcinoma with anaplastic carcinoma are even more infrequent. Herein, we presented a single case (a 17-year-old female) with regular menstrual cycles and stomach pain when eating who was diagnosed with a left ovarian tumor accompanied by mural nodules. The right ovarian cyst, the left ovary, and the fallopian tube were removed by surgery. Intraoperative diagnosis suggested a bilateral ovarian tumor with mural nodules, which include three different pathological types: sarcomatoid transformation, anaplastic carcinoma, and sarcoma. Paclitaxel combined with carboplatin was given for 6 cycles after an operation, and gonadotropin-releasing hormone agonist (GnRHa) was given at the beginning of chemotherapy for 3 cycles for ovarian function protection. Regular follow-up (the last follow-up was performed 48 months after the operation) of gynecological ultrasound and tumor indicators did not indicate recurrence. In clinical practice, it is necessary to pay attention to the symptoms such as abdominal pain in adolescent females. Routine non-invasive pelvic ultrasound is recommended to fully evaluate the nature of the tumor before surgery, and decide the operation mode. Also, intraoperative frozen pathology of the tissue should be performed as soon as possible.

## Background

Ovarian tumors are common tumors of female reproductive organs, frequently diagnosed in childbearing age and perimenopausal period. Adolescent ovarian tumors rarely occur, having an incidence of 2.6/100,000 [1]. The majority of pathological diagnoses are benign, while malignant tumors account for about 20% ~ 30% [2]. Because of the particularity of the ovary in embryogenesis, ovarian tumors have an organizational structure and complex composition. Among different ovarian tumor types, epithelial origin accounts for about 13% of adolescent ovarian tumors [3]. A borderline ovarian tumor (BOT) represents an independent group of ovarian neoplasms with atypical epithelial proliferation, which are very uncommon. Mucinous and serous ovarian tumors are the most common types of BOT, and the incidence of mucinous borderline ovarian tumor (MBOT) is high among the Asian population [4].

Herein, we presented a single case with a left ovarian tumor accompanied by mural nodules. Preoperative diagnosis suggested a bilateral ovarian tumor, rapid intraoperative pathology suggests that mural nodules may contain three different pathological types: sarcomatoid transformation, anaplastic carcinoma, and sarcoma. Anaplastic carcinoma and sarcoma are malignant tumors [5]. This study retrospectively analyzed the clinical and pathological data of the patient and discussed its clinical manifestations, treatment options, and prognosis. We also discussed previous reports published in the literature to improve the understanding of this disease.

## Case presentation

In April 2018, a 17-year-old female was admitted to Nanjing First Hospital. Preoperative diagnosis suggested a bilateral ovarian tumor. She underwent surgery. Postoperative pathological diagnosis suggested the following: (1) left ovarian mucinous borderline tumor with intraepithelial carcinoma with mural anaplastic cancer nodule; (2) right ovarian mucinous cystadenoma.

## Clinical data

The patient had her first menstruation at the age of 13. Since then, she has had regular menstruation accompanied by mild dysmenorrhea. She was unmarried and childless. In the past two years, she experienced occasional pain in the stomach, especially after eating at nightand underwent several gastroscopy examinations. The laboratory indexes revealed CA199 52.47U/mL (the normal value is lower than 27U/ml), CA125 36.08U/mL (the normal value is lower than 35U/ml) and D-dimer 0.74ug/mL (the normal value is lower than 0.5ug/mL). The pelvic ultrasound showed a mixed cystic mass where visible internal separation was found in the anterior upper part of the uterus, the size was 17.4*12*13.3 cm, and a dense point echo was found in part of the cystic cavity, while a middle and high echo of irregular nature were found in part of the cavity. Complete chest and abdominal computed tomography (CT) showed no abnormal density shadow in the retroperitoneum and chest, but there was a huge mass occupying the pelvic and abdominal cavity, which was compressing the bladder and uterine body, with a slightly higher density. There was also a thin liquid shadow, multi-room shape, and smooth wall, with a size of about 17.6*14.3 cm. Inside, there seemed to be a point of dense lamellar shadow, enhanced scanning, and the wall and septum were strengthened.

## Surgical information

Because the malignant lesion of the left ovarian tumor could not be excluded, a bilateral ovarian cyst was removed. Intraoperative probe suggested a few bloody ascites in the pelvic cavity; the uterus was of normal size, the left ovary was enlarged by about 18*15*15 cm, and the 3*2 cm rupture was seen at the tip of the tumor with thick yellowish liquid that was slowly leaking. A yellow parenchyma growth was seen in the capsule wall, which was about 3*3 cm in size. The left fallopian tube extends and attaches to the surface of the mass. Cystic enlargement of the right ovary was about 6*5*4 cm, and the right fallopian tube appearance was normal.

Intraoperative frozen pathological further suggested left ovarian mucinous borderline tumor with mural nodules, which were identified as sarcomatoid, and right ovarian mucinous cystadenoma. Consequently, left ovary and fallopian tube resections were performed.

## The pathologic result

### Macroscopic view

The size of the left ovarian tumor was 13*12*4.5 cm, with a cystic section and a thickness of 0.2–0.5 cm. Multiple nodular projections were seen on the inner wall of the cyst with a maximum diameter of 2.5–4.5 cm. The nodules were multilocular cystic in section with mucous accumulation. One of the nodules, 3.5*3 cm in size, was pale yellow in section, solid, and slightly tough. The size of the right ovarian cyst was 6*4.5*4 cm.

### Microscopic examination

**Fig. 1:**
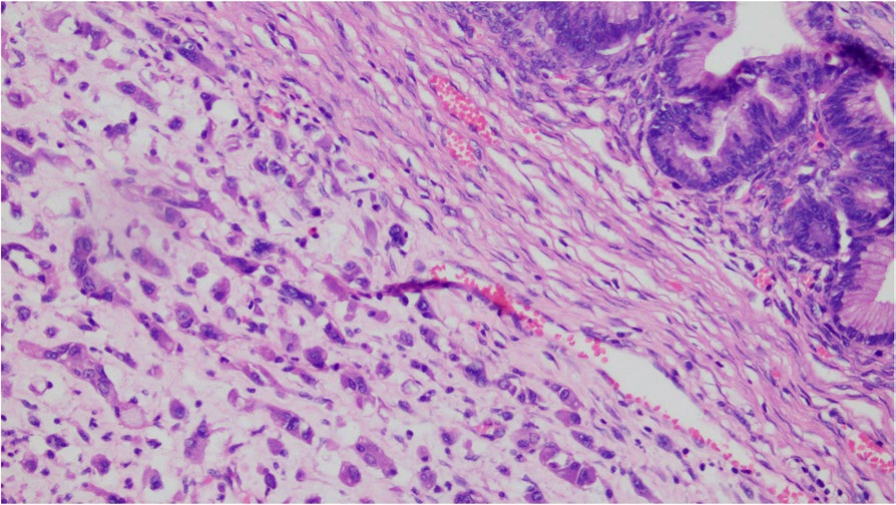
(Mural nodules and epithelial tissue) HE 200

**Fig. 2:**
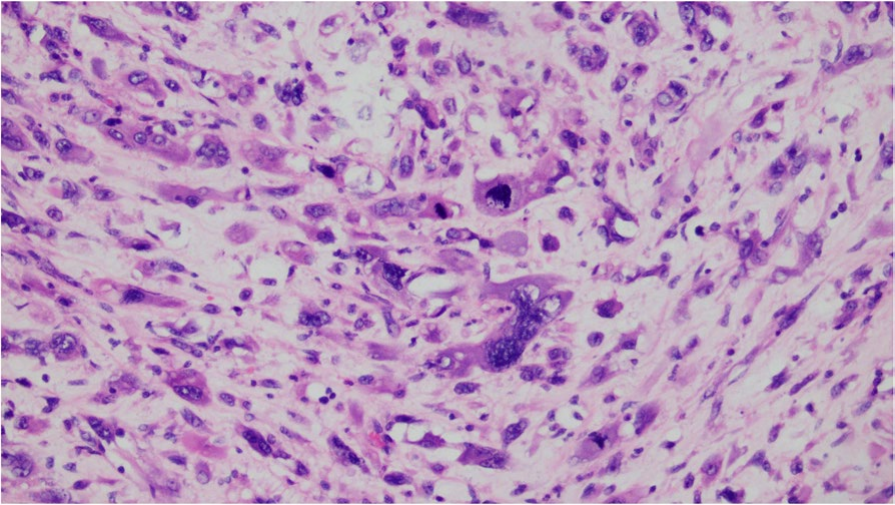
(Mural nodules) HE 200

**Fig. 3:**
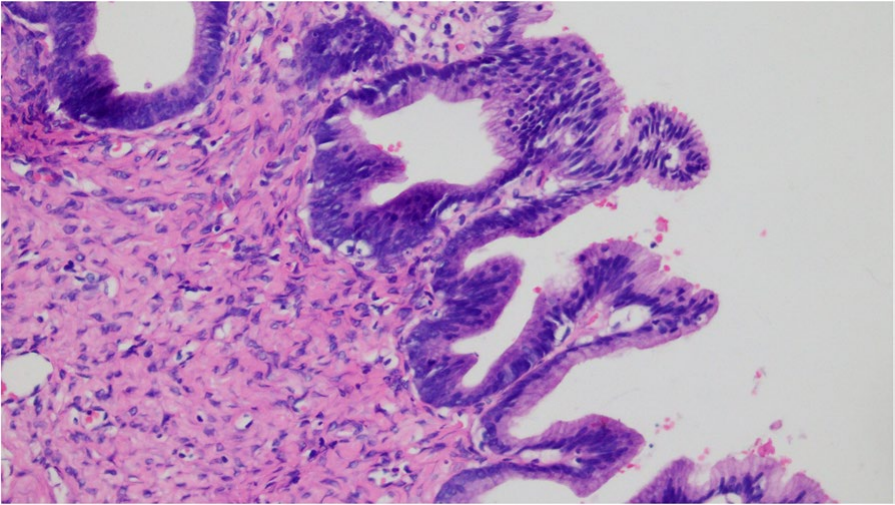
(Ovarian borderline tumor) HE 200

**Fig. 4:**
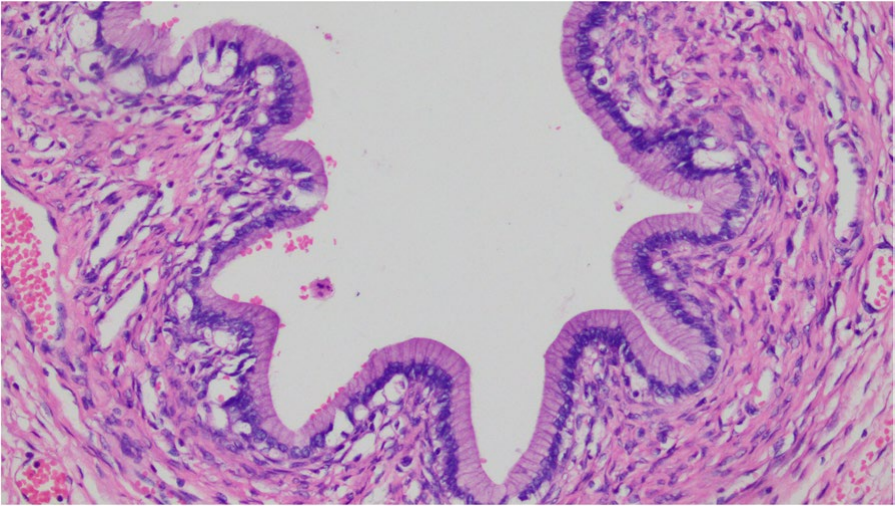
(Ovarian mucinous cystadenoma) HE 200

**Fig. 5:**
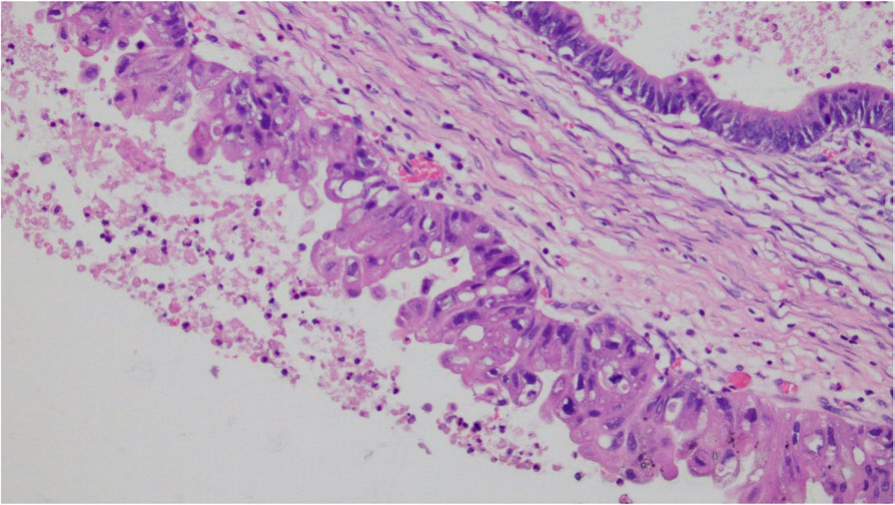
(Ovarian Intraepithelial Carcinoma) HE 200

The cystic wall of the left ovarian mass was partially lined with a single layer of mucinous columnar epithelium. The nucleus was located at the base, and the cytoplasm was rich and full of mucous. The papillary and villous glandular tubular structures were partly lined with a laminated epithelium. The epithelium of small foci was severely heterotic; the nuclei were hyperchromatic and heteromorphic; the polarity was not seen, and the cytoplasm was eosinophilic. The mural nodules were tuberous lesions in the capsule wall. The nodules were arranged in sheets by large polygonal cells or spindle cells, with high-grade nuclei and abundant eosinophilic cytoplasm. There were also scattered giant tumor cells with double or multiple nuclei with obvious nuclear atypia. The cells were arranged in nests or cords (Fig. 1,2,3,4,5).

### Immunohistochemistry

**Fig. 6:**
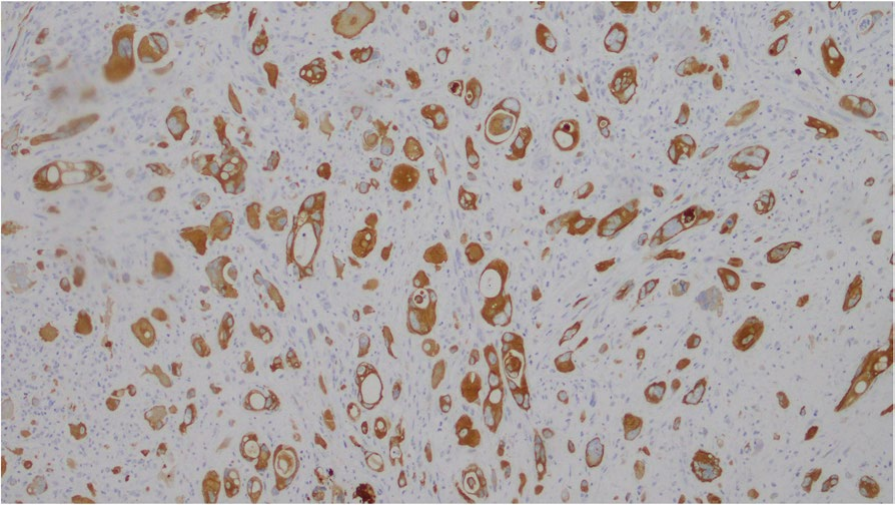
CK

**Fig. 7:**
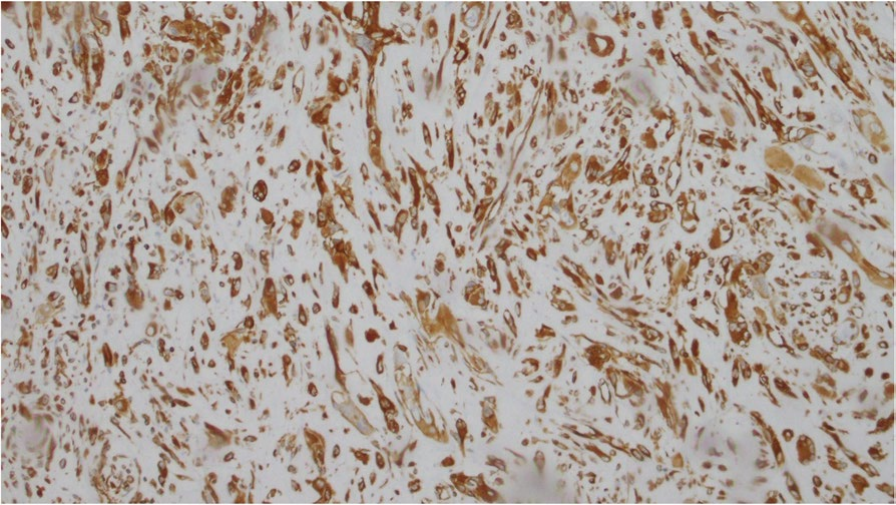
Vim

**Fig. 8:**
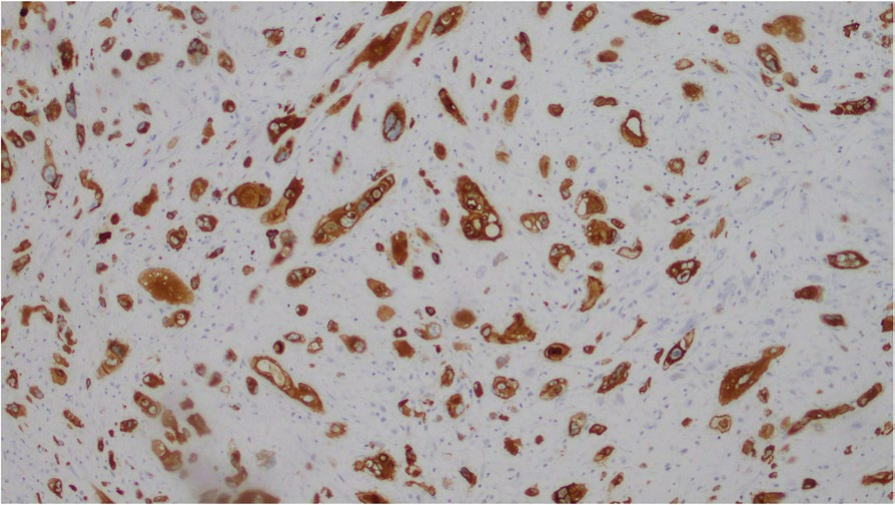
EMA

**Fig. 9:**
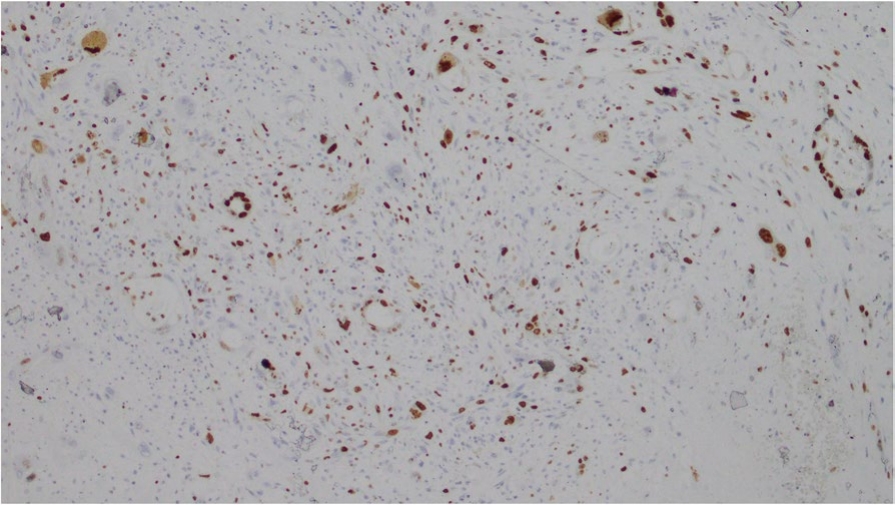
Ki-67

Mural nodules: polygon or spindle and giant tumor cells showed strong positive expression of cytokeratin (CK), epithelial membrane antigen (EMA), and vimentin (VIM), while CD163, S-100, Desmin, CD31, and MyoD1 were negative. Tumor cells expressed SDHB and Ki-67 at about 30% (Fig. 6,7,8,9).

## Postoperative treatment and follow-up

Paclitaxel combined with carboplatin was given for 6 cycles after an operation, and gonadotropin-releasing hormone agonist (GnRHa) was given at the beginning of chemotherapy for 3 cycles for ovarian function protection. Positron emission tomography/computed tomography (PET/CT) and total abdominal CT reexamination after chemotherapy showed no significant abnormalities. The patient was tested for the BRAC1/2 gene, and the germline mutation was negative.

In November 2018, the patient's family required a second supplementary operation to remove the appendix and omentum majus. Intraoperative exploration suggested no obvious pelvic metastasis. The pathological report indicated the appendix tissue, abscess in part of the glandular cavity, and omentum macro momentum and no tumor invasion. Regular follow-up (the last follow-up was performed 48 months after the operation) of gynecological ultrasound and tumor indicators did not indicate recurrence.

## Discussion and conclusion

In 1971, the International Federation of Gynecology and Obstetrics (FIGO) 1971 and the World Health Organization (WHO) characterized borderline epithelial tumors as mucous, serous, transparent cells. Since then, bilateral ovarian epithelial tumors have been rarely reported in adolescents. Abdominal pain is usually the most common clinical symptom, followed by abdominal distension, vaginal bleeding and so on, while surgery is considered the main treatment. Studies have also shown that borderline ovarian tumors are usually non-stromal infiltrates and are mostly surface implants. Unilateral adnexectomy is safe in adolescents with fertility preservation. Recurrence may be seen long after initial diagnosis and treatment; thus, long-term follow-up (over 5 years) is required [6].

The diagnosis of ovarian intraepithelial carcinoma (OIC) depends on the pathological results, presenting with focal or multi-focal cells and/or severe nuclear atypia. In 1999, Riopel et al*.* [7] proposed that OIC was precancerous of ovarian cancer lesion, indicating borderline tumors' progress to carcinogenesis, i.e., from epithelial hyperplasia of cystadenoma to cystadenoma intraepithelial carcinoma of borderline tumor and micro-invasion to infiltrating carcinoma. However, this type is limited to ovarian mucinous tumors. If there is a pseudomyxoma on the peritoneum or bilateral ovarian tumor, the appendix should be removed, and abdominal exploration should be conducted; otherwise, secondary lesions may be found in a short time. OIC is a low-grade malignant tumor. If there is no surrounding invasive implantation, the prognosis is good. However, surgery is recommended to remove the appendix of the affected side. Haeter et al*.* [8] suggested that simple stripping of ovarian cysts was a high-risk factor for disease recurrence. If there are high-risk factors for poor prognoses, such as invasive implantation and micro-nipple, a paclitaxel and platinum-based chemotherapy regimen is suggested.

Previous studies showed more pathological manifestations and immunohistochemical identification of mural nodules as anaplastic carcinoma. Part et al*.* [9] reported 4 cases of ovarian mucinous tumors with anaplastic carcinoma, indicating that the difference in prognosis between sarcomatous nodules and anaplastic carcinoma or sarcoma was the main reason for their differential significance and that sarcomatoid nodules are essentially malignant, which does not affect the prognosis of the disease because of the possibility of small and limited lesions. They concluded that the diagnosis of anaplastic carcinoma was based on unclear nodular boundaries, invasion of blood vessels, and absence of significant inflammatory response in gingival neoplastic polynuclear giant cells; microscopic description of atypical round to oval cells with abundant eosinophilic cytoplasm is consistent with this case. Furthermore, Masi-guiu et al*.* [10] reported a case of 22-year-old female ovarian mucinous cystadenocarcinoma with 5 sarcoma-like mural nodules (SLMN); no gingival neoplastic giant cells were found; immunohistochemical results showed that most cells in nodules were positive for vimentin and cytosine, and negative for epithelial markers such as keratin. In conclusion, SLMN may only be an inflammatory response to bleeding, mucin, and tumor products, without any prognostic significance. Although SLMN may contain a small number of keratin-positive cells, the strong positive and diffuse keratin should be interpreted as evidence of anaplastic carcinoma, and immunohistochemistry, in this case, conforms to the above expression [10, 11]. In recent years, more and more research has been done on the mechanism of the occurrence of mural nodules in anaplastic carcinomas. Currently, there are two versions: dedifferentiated forms and collision tumors. Newer studies suggest that mucinous tumors and mural nodules often share the K-ras gene. Mutational status, mural nodules in anaplastic carcinoma may represent a dedifferentiated form of mucinous neoplasms [12, 13], and mural nodules may develop by acquiring additional genetic alterations (e.g., p53 and PTEN mutations) leading to an anaplastic morphological phenotype [14]. Yamazaki et al*.* [15] proposed that the occurrence of mural nodules may be related to the possible presence of totipotent stem cells in the stroma of ovarian mucinous tumors.

From the initial differential diagnosis to the current mechanism exploration, the clinical diagnosis and treatment of rare cases of the ovarian mucinous tumor with anaplastic carcinomatosis tend to eventually improve. Of course, the discussion of disease prognosis is particularly important. Although anaplastic carcinoma has a high degree of malignancy, it is unknown whether this component in the mural nodules contributes to the rapid progression of the disease. In 2008, Provenza et al*.* [16] suggested no adverse outcomes in the lesions of amt in early unruptured ovarian mucinous tumors, and the prognosis was mainly dependent on the tumor itself. For years, studies have been considering the stage of ovarian tumor and spontaneous rupture key factors affecting the prognosis. However, in 2016, Mhawech-Fauceglia et al*.* [17] reported a case of a 36-year-old female with Ia stage mucinous borderline tumor with anaplastic carcinoma microlesion. Metastatic lesions were found 3 months after surgery, and the patients died of fulminant disseminated intravascular coagulation soon after diagnosis. Therefore, it is necessary to comprehensively evaluate various factors, including the staging of the tumor, metastatic status, etc. Currently, the presence of anaplastic carcinoma mural nodules in unruptured stage I ovarian mucinous tumors does not necessarily lead to a poor prognosis [18, 19].

Ovarian mucinous carcinoma with anaplastic carcinoma lesions in adolescent females has already been reported [20]. In the present study, we reported the first bilateral ovarian epithelial tumor, with one side borderline with intraepithelial carcinoma, which included mural anaplastic carcinoma nodules, and has not yet been reported at home and abroad, and it is worthy of record and standardized follow-up. In this case, the menstrual cycle was restored after chemotherapy, and the follow-up pelvic ultrasound showed follicular development. Our patient presented with abdominal discomfort, which lasted for 2 years. At that time, she was misdiagnosed with gastritis, and she went to the gynecology department because of sudden and persistent abdominal pain. Thus, abdominal pain, as well as the presence to gastritis and appendicitis, may all be indicators of ovarian neoplasms in adolescent women. Also, routine non-invasive pelvic ultrasound is recommended to fully evaluate the nature of the tumor before surgery and decide on the operation mode. For further surgery, preoperative abdominal CT and gynecological-related tumor indicators should be checked to evaluate the nature of the tumor fully and then decide whether the surgical method is transabdominal or laparoscopic. It is still quite challenging to draw conclusions about the prognosis of this type of disease based on the available research, while the mechanism of occurrence and development still remains unclear. However, the identification of pathological results, the selection of surgical methods, and the establishment of follow-up systems have been gradually clarified and improved. Protection cannot be ignored [21, 22]. Clinicians should ensure the safety of young women's lives and try to protect their fertility as much as possible.

## Data Availability

All data generated or analysed during this study are included in this published article.

## Abbreviations

BOT: Borderline Ovarian Tumor
MBOT: Mucinous Borderline Ovarian Tumor
OIC: Ovarian Intraepithelial Carcinoma
CT: Computed Tomography
CK: Cytokeratin
EMA: Epithelial Membrane Antigen
VIM: Vimentin
GnRHa: Gonadotropin Releasing Hormone Agonist
PET/CT: Positron Emission Tomography/Computed Tomography
FIGO: The International Federation of Gynecology and Obstetrics
WHO: The World Health Organization
